# Mevastatin-Induced AP-1-Dependent HO-1 Expression Suppresses Vascular Cell Adhesion Molecule-1 Expression and Monocyte Adhesion on Human Pulmonary Alveolar Epithelial Cells Challenged with TNF-α

**DOI:** 10.3390/biom10030381

**Published:** 2020-03-01

**Authors:** Chuen-Mao Yang, Chih-Chung Lin, Chien-Chung Yang, Rou-Ling Cho, Li-Der Hsiao

**Affiliations:** 1 Department of Pharmacology, College of Medicine, China Medical University, Taichung 40402, Taiwan; royeariel760918@gmail.com (R.-L.C.); lidesiao@livemail.tw (L.-D.H.); 2 Department of Post-Baccalaureate Veterinary Medicine, College of Medical and Health Science, Asia University, Wufeng, Taichung 41354, Taiwan; 3 Department of Anesthetics, Chang Gung Memorial Hospital at Linkuo, Kwei-San, Tao-Yuan 33302, Taiwan; chihchung@adm.cgmh.org.tw; 4 Department of Traditional Chinese Medicine, Chang Gung Memorial Hospital at Tao-Yuan, Kwei-San, Tao-Yuan 33302, Taiwan; r55161@cgmh.org.tw; 5 School of Traditional Chinese Medicine, College of Medicine, Chang Gung University, Kwei-San, Tao-Yuan 33302, Taiwan

**Keywords:** mevastatin, HO-1, NF-κB, VCAM-1, c-Jun/AP-1, human

## Abstract

Mevastatin (MVS) has been previously shown to induce heme oxygenase (HO)-1 expression through Nox/ROS-dependent PDGFRα/PI3K/Akt/Nrf2/ARE axis in human pulmonary alveolar epithelial cells (HPAEpiCs). However, alternative signaling pathways might involve in MVS-induced HO-1 expression. We found that tumor necrosis factor α (TNFα) induced vascular cell adhesion protein 1 (VCAM-1) expression and NF-κB p65 phosphorylation which were attenuated by pretreatment with MVS via up-regulation of HO-1, determined by Western blot and real-time qPCR. TNFα-induced VCAM-1 expression was attenuated by an NF-κB inhibitor, Bay117082. The inhibitory effects of MVS were reversed by tin protoporphyrin (SnPP)IX (an inhibitor of HO-1 activity). In addition, pretreatment with the inhibitor of pan-Protein kinase C (PKC) (GF109203X), PKCα (Gö6983), Pyk2 (PF431396), p38α MAPK (SB202190), JNK1/2 (SP600125), or AP-1 (Tanshinone IIA), and transfection with their respective siRNAs abolished MVS-induced HO-1 expression in HPAEpiCs. c-Jun (one of AP-1 subunits) was activated by PKCα, Pyk2, p38α MAPK, and JNK1/2, which turned on the transcription of the *homx1* gene. The interaction between c-Jun and HO-1 promoter was confirmed by a chromatin immunoprecipitation (ChIP) assay, which was attenuated by these pharmacological inhibitors. These results suggested that MVS induces AP-1/HO-1 expression via PKCα/Pyk2/p38α MAPK- or JNK1/2-dependent c-Jun activation, which further binds with AP-1-binding site on HO-1 promoter and suppresses the TNFα-mediated inflammatory responses in HPAEpiCs. Thus, upregulation of the AP-1/HO-1 system by MVS exerts a potentially therapeutic strategy to protect against pulmonary inflammation.

## 1. Introduction

The pathogenesis of immune-mediated inflammatory diseases is associated with pro-inflammatory cytokines such as tumor necrosis factor α (TNFα) [1]. Moreover, TNFα-triggered inflammation is mediated through interaction with some receptors that initiate various intracellular signaling pathways and transcription factors such as NF-κB p65 [2]. Activated NF-κB p65 leading to transcription of inflammatory genes is integrated to the pathogenesis of various chronic diseases [3]. Therefore, development of therapeutic compounds protecting against NF-κB p65-induced inflammatory responses might be a strategy for treating pulmonary diseases. Recent studies indicate that overexpression of heme oxygenase (HO)-1 inhibits the activation of NF-κB p65 to reduce liver fibrosis, intestinal barrier, pulmonary inflammation, and apoptosis [4,5,6].

HO, including three isoforms of HO-1, HO-2, and HO-3, has been known as the rate-limiting enzyme of heme catabolism which converts heme into carbon monoxide (CO), biliverdin, and Fe^2+^ [7]. HO-1 has a low level of expression in normal tissues, which is induced by redox stress or various mediators and exerts protective and anti-inflammatory effects [8]. Several studies have extensively investigated the cytoprotective effects of HO-1 against oxidative injury and cellular stresses [9]. Therefore, purposing old drugs or development of new drugs induced the expression of HO-1 might be beneficial to protect against the inflammatory diseases. Mevastatin (MVS) is a member of the statins, which has been shown to upregulate HO-1 expression and provide an anti-inflammatory property [10]. Our previous study has also proved that MVS plays a protective role by attenuating the COX2/PGE_2_ expression and cell migration challenged by sphingosine-1-phosphate (S1P) in human tracheal smooth muscle cells (HTSMCs) [11]. According to these lines of evidence, we investigated the cytoprotective effects of MVS and elucidated the mechanisms underlying MVS-induced HO-1 expression in human pulmonary alveolar epithelial cells (HPAEpiCs).

Protein kinase C (PKC)α is a member of the classical PKCs that takes a part in the regulation of cellular functions [12]. Upregulation of HO-1 via activation of PKCα could enhance fusion proteins and ameliorate mitochondrial injury [13]. Our previous report also indicated that rosiglitazone (a PPARγ agonist) increases HO-1 expression through the PKCα signaling pathway in HPAEpiCs [6]. Furthermore, non-receptor tyrosine kinases such as proline-rich tyrosine kinase (Pyk2), are one kind of the critical downstream molecules of PKC [14]. Lin et al. found that pretreatment with Pyk2 inhibitor (PF431396) or down-regulation of Pyk2 gene can abolish HO-1 expression [15]. Activation of Pyk2 and PKCα regulated MAPKs (p38 MAPK, JNK1/2, and Erk1/2) signal pathways, leading to HO-1 protein expression [16]. Our previous research showed that p38 MAPK phosphorylation might increase HO-1 level to attenuate adhesion molecules expression induced by lipopolysaccharide (LPS) [6]. The phosphorylation of JNK1/2 has been associated with AP-1 (c-Jun and c-Fos) activity and HO-1 expression under various conditions [16,17,18]. Furthermore, administration of statins induces HO-1 gene expression though p38 MAPK/AP-1 signal pathway [10]. Several lines of evidence indicate that activation of AP-1 stimulates *homx1* gene expression by the various inducers [19,20]. In addition, we have also found that upregulation of AP-1/HO-1 expression suppresses the IL-1β-induced MMP-9 expression and cell migration in brain astrocytes [16]. However, whether activation of PKCα, Pyk2, MAPKs, or AP-1 is involved in the MVS-induced HO-1 expression remained to be elucidated. To approach the hypothesis, we investigated the mechanisms by which MVS induced HO-1 expression through activation of intracellular signaling pathways and suppressing NF-κB p65 and VCAM-1 expression in HPAEpiCs challenged with TNFα.

Although MVS has been extensively used for the treatment of hyperlipidemia, the effects of MVS on lung inflammatory diseases have not been thoroughly evaluated. In particular, the detailed mechanisms involved in MVS-induced HO-1 expression are not completely defined in HPAEpiCs. Our results showed that MVS-enhanced HO-1 expression attenuated the TNFα-induced p65 phosphorylation and VCAM-1 expression in HPAEpiCs. Here, we demonstrated that MVS-induced HO-1 expression is mediated through PKCα/Pyk2/p38 MAPK and JNK1/2-regulated phosphorylation of c-Jun-dependent AP-1 activation and cytoprotective against the TNFα-mediated inflammatory responses in the pulmonary system.

## 2. Materials and Methods

### 2.1. Reagents and Chemicals

Dulbecco’s modified Eagle’s medium (DMEM)/F-12 and fetal bovine serum (FBS) were from Invitrogen (Carlsbad, CA, USA). GenMute™ siRNA Transfection Reagent was from SignaGen Laboratories (Rockville, MD, USA). Mevastatin (MVS), Bay 11-7082, Gö6983, SP600125, and tin protoporphyrin IX (SnPPIX) were from Cayman Chemical (Ann Arbor, MI, USA). PF431396 was from Merck (Billerica, MA, USA). Anti- glyceraldehyde 3-phosphate dehydrogenase (GAPDH) (MCA-1D4) antibody was from EnCor Biotechnology (Gainesville, FL, USA). Anti-VCAM-1[EPR50381(2)] (ab174279), anti-NF-κB p65 (phospho-S^536^) (ab86299), anti-PKCalpha (phospho-S^657^) [EPR1901(2)] (ab180848), anti-Pyk2 [E354] (ab32448), and anti-c-Jun [E254] (ab32137) were from Abcam (Cambridge, UK). GF109203, SB202190, Tanshinone IIA, and anti-HO-1 pAb (ADI-SPA-895) were from Enzo Life Sciences (Farmingdale, NY, USA). Anti-β-actin (C4) (sc-47778), anti-PKCα (C20) (sc-208), anti-Gαs (K20) (sc-823), and anti-JNK1/2 (E5) (sc-137020) antibodies were from Santa Cruz Biotechnology (Santa Cruz, CA, USA). Anti-NF-κB p65 (D14E12) XP (#8242), anti-phospho-Pyk2 (Tyr^402^) (#3291), anti-p38 MAPK (D13E1) XP (#8690), anti-phospho-c-Jun (Ser^63^) (#2361), anti-phospho-SAPK/JNK(Thr^183^/Tyr^185^) (#9255), and anti-phospho-p38 MAPK (Thr^180^/Tyr^182^) (#9211) were from Cell Signaling Technology (Danvers, MA, USA). TRIZOL, 2,3-bis-(2-methoxy-4-nitro-5-sulfophenyl)-2H-tetrazolium-5-carboxanilide (XTT) assay kit, and other chemicals were from Sigma (St. Louis, MO, USA).

### 2.2. Cell Culture and Treatment

HPAEpiCs were purchased from the ScienCell Research Laboratories (San Diego, CA, USA) and cultured in DMEM/F12 medium containing 10% FBS at 37 °C in a humidified 5% CO_2_. Experiments were performed with cells from passages 4 to 7, as previously described [21].

### 2.3. Protein Preparation and Western Blot Analysis

Growth-arrested cells were incubated with or without 30 μM MVS at 37 °C for the indicated time periods. Inhibitors were added 1 h prior to the application of MVS, as previously described [21]. In brief, the cells were washed with cold PBS, scraped, and collected with a lysis buffer (50 mM Tris-HCl, pH 7.4, 1 mM EGTA, 1 mM NaF, 150 mM NaCl, 1 mM PMSF, 5 μg/mL leupeptin, 20 μg/mL aprotinin, 1 mM Na3VO4, 1% Triton). A BCA reagent was used to determine the levels of protein concentration. Each sample was added by the x5 sample buffer to the same protein concentration. The same amounts of protein (30 μg) were denatured and analyzed by 10% SDS-PAGE. Then, the nitrocellulose membranes that protein was transferred to were probed overnight with respective primary antibodies. Membranes were washed with Tris-Tween 20 buffered saline (TTBS) four times for 5 min each and incubated with anti-rabbit or anti-mouse horseradish peroxidase antibody (1:2000 dilution) for 1 h. Finally, the immunoreactive bands were detected by ECL and captured using a UVP BioSpectrum 500 Imaging System (Upland, CA, USA). The UN-SCAN-IT gel software (Orem, UT, USA) was used to quantify image densitometry analysis. All image densitometry analyses were normalized to β-actin or total protein.

### 2.4. Real-Time Quantitative PCR (RT-qPCR) Analysis

TRIzol reagent was used to extract the total RNA from HPAEpiC that was spectrophotometrically determined at 260 nm as previously described [21]. In brief, mRNA was reverse-transcribed into cDNA and analyzed by RT-qPCR. RT-qPCR was performed with a StepOnePlus^TM^ real-time qPCR system (ThermoScientific-Applied Biosystems, Foster City, CA, USA) and Kapa Probe Fast qPCR Kit Master Mix (2X) Universal (KK4705; KAPA Biosystems, Wilmington, MA, USA). The levels of HO-1 and VCAM-1 expression were quantified by normalization to the level of GAPDH expression. The ΔΔ^Ct^ method was used to determine relative gene expression, where Ct represented the threshold cycle. All experiments were performed in triplicate.

### 2.5. Transient Transfection with siRNAs in HPAEpiCs

Human siRNAs of scrambled, SMARTpool RNA duplexes corresponding to PKCα (SASI_Hs01_00018816), Pyk2 (SASI_Hs01_00032249), JNK1 (SASI_Hs02_00319556), and scrambled control (negative control type 1) siRNA were from Sigma-Aldrich (St. Louis, MO, USA). JNK2 (HSS108550), p38α (HSS102352, HSS102353, HSS175313), and c-Jun (HSS105641, HSS105642, HSS180003) were from Invitrogen Life Technologies (Carlsbad, CA, USA). Briefly, transient transfection of siRNAs was carried out by using Opti-MEM and Genmute reagent. The transfection complex (siRNA 100 nM, Opti-MEM 100 μL, and Genmute reagent 2.5 μL) was directly added to the cells and incubated for 5 h. The cells were replaced with DMEM/F-12 medium containing 10% FBS for overnight and then changed to serum free medium for 48 h. The sequences of siRNAs were listed as below:

Scrambled: 5′-UUCUCCGAACGUGUCACGU-3′,
PKCα: 5′-AUAAGGAUCUGAAAGCCCGUUUGGA-3′,
Pyk2: 5′-CUGAUGACCUGGUGUACCU-3′,
JNK1: 5′-GCAGAAGCAAGCGUGACAACA-3′,
JNK2: 5′-AAUUGGUUUCAGCUGGUAACGU-3′,
p38α:
  (1) 5′-AUGAAUGAUGGACUGAAAUGGUCUG-3′,
  (2) 5′-AAACAAUGUUCUUCCAGUCAACAGC-3′,
  (3) 5′-UUAGGUCCCUGUGAAUUAUGUCAGC-3′,
c-Jun:
  (1) 5′-AAGUUGCUGAGGUUUGCGUAGACCG-3′,
  (2) 5′-AACUGCUGCGUUAGCAUGAGUUGGC-3′,
  (3) 5′-AUAGAAGGUCGUUUCCAUCUUUGCA-3′.

### 2.6. Isolation of Subcellular Fractions

The cells were incubated with MVS for the indicated time intervals. Phorbol 12-myristate 13-acetate (PMA) was a PKCα activator which was used as a positive control. The cytosolic and membrane fractions were isolated according to the procedure, as described previously [22].

### 2.7. Chromatin Immunoprecipitation (ChIP) Assay

ChIP assay was performed to detect the association of transcription factors with the human HO-1 promoter, as previously described [21]. Soluble chromatin was prepared using a ChIP assay kit (Upstate, Lake Placid, New York, NY, USA) according to the instructions of the manufacturer and immunoprecipitated without (control) or with an anti-c-Jun antibody and normal goat immunoglobulin G (IgG). PCR products for all SYBR Green primer pairs were verified to produce single products by high-resolution melt curve for avoiding the possibility of amplification artifacts. The comparative Ct method (ΔΔ^Ct^) was used to calculate the relative mRNA levels. The DNA was extracted and resuspended in H_2_O and subjected to PCR amplification with the ARE primers: AP-1 (444 bp), F: 5′-AGAGC CTGGG GTTGC TAAGT-3′ and R: 5′-GGCCG GTCAC ATTTA TGCTC-3′.

### 2.8. Data and Statistical Analysis

GraphPad Prism Program 6.0 software (GraphPad, San Diego, CA, USA) was used to perform statistical analysis. We used one-way ANOVA followed by Dunnett’s post hoc test when comparing more than two groups of data as previously described [21]. *p*-values of 0.01 were considered to be statistically significant. Post hoc tests were analyzed only if F achieved *p* < 0.01 and there was not significant in the homogeneity of variance. Error bars were omitted when they fell within the dimensions of the symbols. All the data were expressed as the mean ± SEM, at least five individual experiments (*n* = 5).

## 3. Results

### 3.1. Up-Regulation of HO-1 by MVS Reduces the TNFα-Induced VCAM-1 Expression

**Figure 1:**
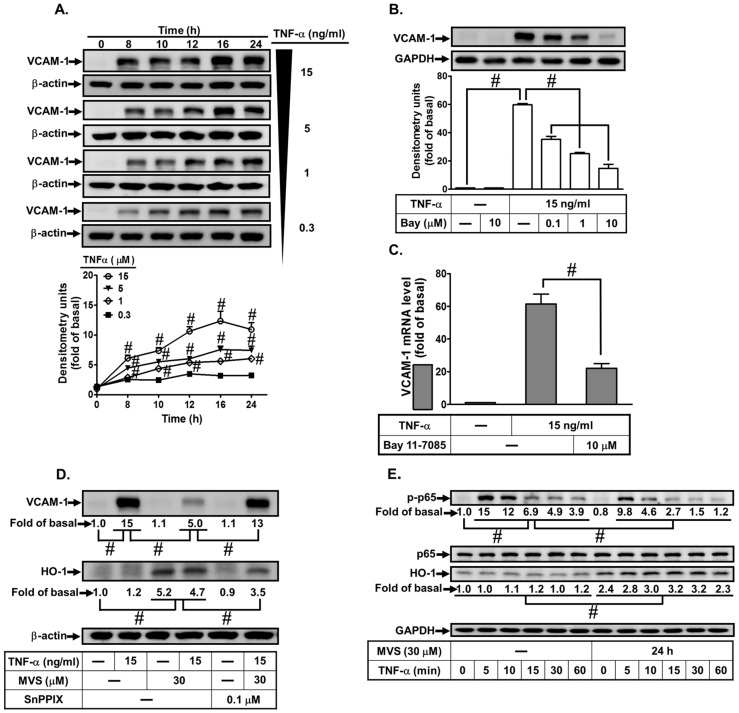
Mevastatin (MVS)-induced heme oxygenase (HO)-1 expression attenuates tumor necrosis factor α (TNFα)-stimulated phosphorylation of NF-κB p65 and VCAM-1 expression. (**A**) Human pulmonary alveolar epithelial cells (HPAEpiCs)s were incubated with different concentrations of TNFα (15, 5, 1, or 0.3 ng/mL) for the indicated time intervals. (**B**,**C**) Cells were pretreated different concentrations of Bay11-7082 (0.1, 1, or 10 μM) for 1 h, and then incubated with or without TNFα (15 ng/mL) for 24 h (protein) or 4 h (mRNA). (**D**) HPAEpiCs were pretreated with 30 μM MVS for 1 h, incubated with or without tin protoporphyrin IX (SnPPIX) (0.1 μM) for 1 h, and then stimulated with TNFα for 24 h. (**E**) Cells were pretreated with 30 μM MVS for 24 h and then incubated with TNFα (15 ng/mL) for the indicated time intervals. (**A**,**B**,**D**,**E**) The levels of VCAM-1, β-actin, glyceraldehyde 3-phosphate dehydrogenase (GAPDH), phospho-p65, p65, and HO-1 protein were determined by Western blot using respective antibodies as indicated. Data are expressed as mean ± SEM of five independent experiments (*n* = 5). ^#^ *p* < 0.01, as compared with the cells exposed to the indicated reagents.

Our previous study shows that up-regulation of HO-1 protects against lung inflammatory responses induced by LPS [21]. In addition, MVS has been shown to time-dependently induce the expression of HO-1 expression in HPAEpiCs [23]. Here, we evaluated the effect of HO-1 expression by MVS on the TNF-α-induced VCAM-1 expression in HPAEpiCs. First, TNF-α induced VCAM-1 protein and mRNA expression in concentration- and time-dependent manners (Figure 1A,C). NF-κB p65 phosphorylation has been shown to be a key player in the expression of various inflammatory mediators resulting in pulmonary diseases [24]. Here, we found that TNFα-induced VACM-1 expression was reduced by an NF-κB inhibitor Bay11-7082 (Figure 1B,C). Up-regulation of HO-1 by MVS attenuated the TNFα-induced VCAM-1 expression and phosphorylation of NF-κB p65 (Figure 1D,E), which was reversed by an HO-1 inhibitor tin protoporphyrin IX (SnPPIX) (Figure 1D). These results suggested that MVS-upregulated HO-1 expression attenuates VCAM-1 expression in HPAEpiCs challenged with TNFα through suppressing the activation of NF-κB p65.

### 3.2. PKCα Is Required for MVS-Induced HO-1 Expression

**Figure 2:**
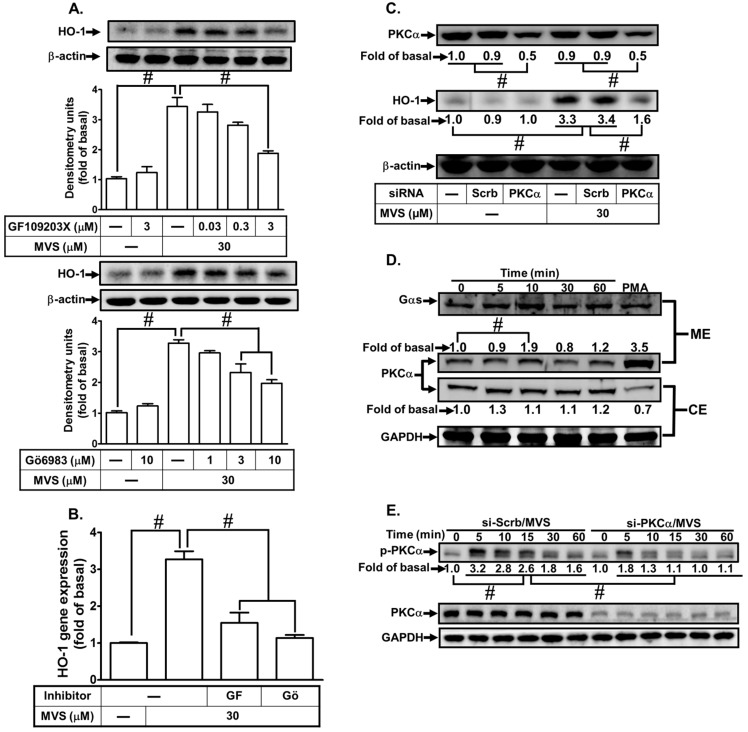
Protein kinase C (PKC)α is involved in MVS-induced HO-1 expression. (**A**) Cells were pretreated with various concentrations of GF109203X or Gö6983 for 1 h, and then incubated with vehicle or MVS (30 μM) for 24 h. The levels of HO-1 and β-actin protein were determined by Western blot. (**B**) The cells were pretreated with GF109203X (3 μM) or Gö6983 (10 μM) for 1 h and then incubated with vehicle or MVS (30 μM) for 8 h. The levels of HO-1 mRNA were determined by real-time qPCR. (**C**) HPAEpiCs were transfected with scrambled (Scrb) or PKCα siRNA, and then incubated with MVS for 24 h. The levels of HO-1, PKCα, or β-actin were determined by Western blot. (**D**) Cells were pretreated with MVS (30 μM) for the indicated time intervals. The cytosol and membrane fractions were prepared and analyzed by Western blot using an anti-PKCα, anti-GAPDH, or anti-Gαs antibody. (**E**) HPAEpiCs were transfected with scrambled (Scrb) or PKCα siRNA and then incubated with vehicle or MVS (30 μM) for the indicated time intervals. The levels of phospho-PKCα, PKCα, and GAPDH were determined by Western blot using respective antibodies. Data are expressed as mean ± SEM of five independent experiments (*n* = 5). ^#^ *p* < 0.01, as compared with the cells exposed to the indicated reagents.

Our previous study has shown that activation of PKCα involves in upregulation of HO-1 by rosiglitazone in HPAEpiCs [6]. Here, we investigated whether PKCα is also involved in the MVS-induced HO-1expression. We found that pretreatment with the inhibitors of non-selective PKC (GF109203X) or selective PKCα (Gö6983) concentration-dependently attenuated the MVS-induced HO-1 protein and mRNA expression in HPAEpiCs (Figure 2A,B). Further, to ensure the role of PKCα in the MVS-stimulated HO-1 protein expression, as shown in Figure 2C, transfection with PKCα siRNA reduced the PKCα protein level and attenuated the MVS-induced HO-1 expression. Activation of PKCα is translocated from the cytosolic to the membrane fraction in response to various stimuli [25]. As expectedly, we observed that MVS stimulated PKCα translocation from the cytosolic to the membrane fraction within 10 min (Figure 2D). We further investigated whether MVS-stimulated HO-1 expression is mediated through phosphorylation of PKCα. MVS time-dependently stimulated phosphorylation of PKCα which was reduced by transfection with PKCα siRNA (Figure 2E). These results suggested that MVS-induced HO-1 expression is, at least in part, mediated through phosphorylation of PKCα in HPAEpiCs.

### 3.3. Involvement of Pyk2 in MVS-Induced HO-1 Expression

**Figure 3:**
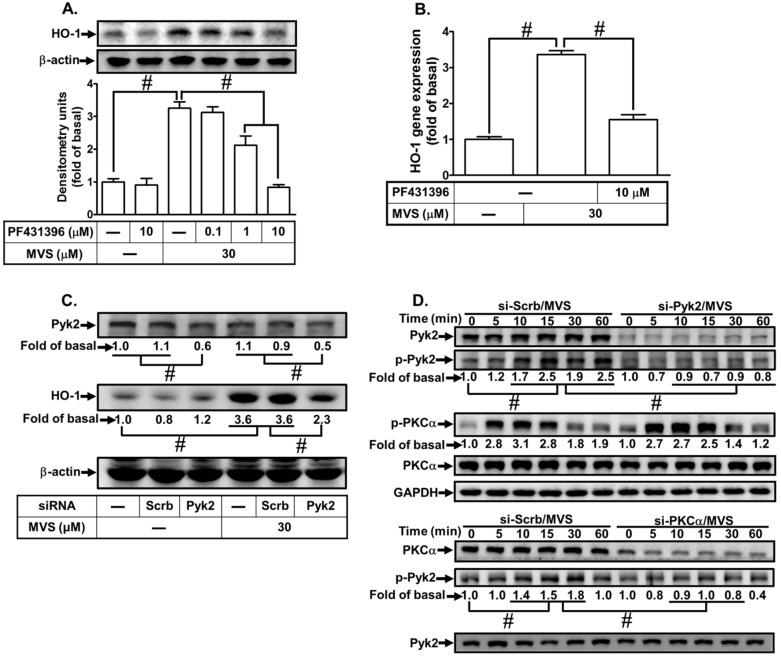
Phosphorylation of Pyk2 is involved in the MVS-induced HO-1 expression. (**A**) HPAEpiCs were pretreated with various concentrations of PF431396 for 1 h, and then incubated with vehicle or MVS (30 μM) for 24 h. The levels of HO-1 and β-actin protein expression were determined by Western blot. (**B**) The cells were pretreated with PF431396 (10 μM) for 1 h and then incubated with vehicle or MVS (30 μM) for 8 h. The levels of HO-1 mRNA were analyzed by real-time qPCR. (**C**) HPAEpiCs were transfected with scrambled (Scrb) or Pyk2 siRNA, and then incubated with MVS for 24 h. The levels of Pyk2, HO-1, and β-actin protein expression were determined by Western blot. (**D**) HPAEpiCs were transfected with PKCα or Pyk2 siRNA and then treated with MVS for the indicated time intervals. The levels of phospho-PKCα, PKCα, phospho-Pyk2, Pyk2, and GAPDH were determined by Western blot using respective antibodies. Data are expressed as mean ± SEM of five independent experiments (*n* = 5). ^#^ *p* < 0.01, as compared with the cells exposed to the indicated reagents.

PKC has been shown to activate various downstream signaling components including Pyk2 [14]. Thus, we investigate whether Pyk2 is involved in the MVS-induced HO-1 expression in HPAEpiCs, a Pyk2 inhibitor PF431396 was used for this purpose. We found that pretreatment with PF431396 concentration-dependently reduced the MVS-induced HO-1 protein and mRNA expression (Figure 3A,B). Further, to confirm the role of Pyk2 in MVS-mediated signal responses, as shown in Figure 3C, transfection with Pyk2 siRNA knocked down the level of Pyk2 protein and then reduced the MVS-induced HO-1 expression. We also determined whether phosphorylation of Pyk2 participated in the MVS-induced HO-1 expression in HPAEpiCs. As shown in Figure 3D, MVS time-dependently stimulated the phosphorylation of Pyk2 which was attenuated by transfection with Pyk2 siRNA. Pyk2 siRNA failed to change the phosphorylation of PKCα. In addition, transfection with PKCα siRNA reduced MVS-stimulated phosphorylation of Pyk2, suggesting that Pyk2 is a downstream component of a PKCα-dependent pathway. These results suggested that MVS-induced HO-1 expression is mediated through a PKCα/Pyk2 cascade in HPAEpiCs.

### 3.4. MVS-Induced HO-1 Expression Is Mediated through p38 MAPK and JNK1/2

**Figure 4:**
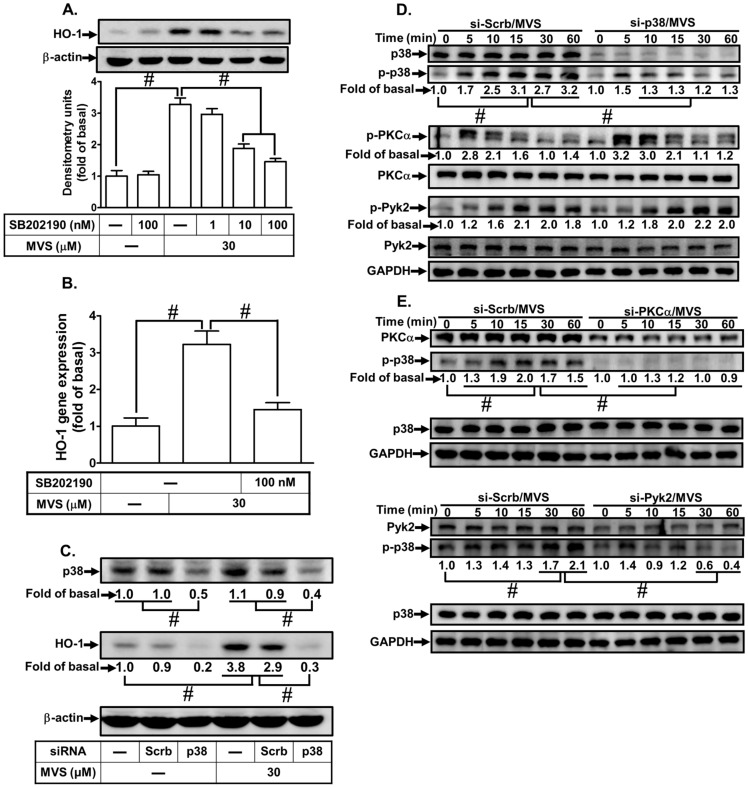
p38 MAPK is involved in the MVS-induced HO-1 expression. (**A**) HPAEpiCs were pretreated with various concentrations of SB202190 for 1 h, and then incubated with vehicle or MVS (30 μM) for 24 h. The levels of HO-1 and β-actin protein expression were determined by Western blot. (**B**) The cells were pretreated with SB202190 (100 nM) for 1 h and then incubated with MVS for 8 h. The levels of HO-1 mRNA were analyzed by real-time qPCR. (**C**) Cells were transfected with scrambled (Scrb) or p38 MAPK siRNA, and then incubated with MVS for 24 h. The levels of p38 MAPK, HO-1, and β-actin protein expression were determined by Western blot. (**D**,**E**) HPAEpiCs were transfected with scrambled (Scrb), PKCα, Pyk2, or p38 MAPK siRNA and then incubated with MVS for the indicated time intervals. The levels of phospho-p38 MAPK, p38 MAPK, phospho-PKCα, PKCα, phospho-Pyk2, Pyk2, and GAPDH were determined by Western blot using respective antibodies. Data are expressed as mean ± SEM of five independent experiments (*n* = 5). ^#^ *p* < 0.01, as compared with the cells exposed to the indicated reagents.

Up-regulation of HO-1 by MAPKs could play a protective role in suppressing inflammatory responses and cell death induced by various insults [6,10,26]. First, we investigated whether p38 MAPK is involved in HO-1 expression. Our results showed that pretreatment with the inhibitor of p38 MAPK (SB202190) attenuated the MVS-induced HO-1 protein (Figure 4A) and mRNA (Figure 4B). Further, to confirm the role of p38 MAPK in the MVS-induced HO-1 expression, transfection of HPAEpiCs with p38 MAPK siRNA knocked down the level of p38 MAPK protein and reduced the MVS-induced HO-1 expression (Figure 4C). In addition, increased phosphorylation of p38 MAPK could activate the induction of HO-1 [27]. To confirm whether MVS-stimulated phosphorylation of p38 MAPK involved in HO-1 expression, as shown in Figure 4D, transfection with p38 MAPK siRNA down-regulated the level of p38 MAPK protein and attenuated phosphorylation of p38 MAPK, but not PKCα or Pyk2. In addition, MVS-stimulated p38 MAPK phosphorylation was attenuated by transfection with either PKCα or Pyk2 siRNA (Figure 4E). These results suggested that MVS-induced HO-1 expression is mediated through PKCα/Pyk2/p38α MAPK in HPAEpiCs.

**Figure 5:**
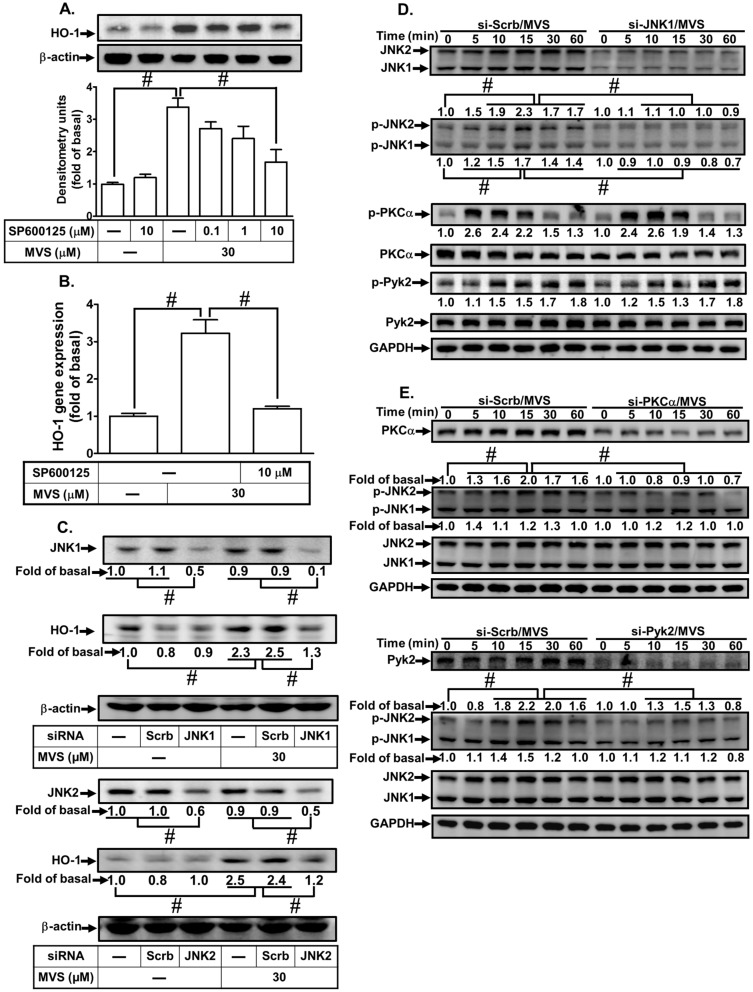
Activation of JNK1/2 contributes to the MVS-induced HO-1 expression. (**A**) HPAEpiCs were pretreated with various concentrations of SP600125 for 1 h and then incubated with MVS for 24 h. The protein levels of HO-1 and β-actin were determined by Western blot. (**B**) Cells were pretreated with SP600125 (10 μM) for 1 h and then incubated with MVS for 8 h. The levels of HO-1 mRNA were determined by real-time qPCR. (**C**) Cells were transfected with scrambled (Scrb), JNK1, or JNK2 siRNA and then incubated with MVS for 24 h. The levels of JNK1, JNK1, HO-1, and β-actin protein expression were determined by Western blot. (**D**,**E**) HPAEpiCs were transfected with scrambled (Scrb), PKCα, Pyk2, or JNK1/2 siRNA and then incubated with MVS for the indicated time intervals. The levels of phospho-JNK1/2, JNK1/2, phospho-PKCα, PKCα, phospho-Pyk2, Pyk2, or GAPDH were determined by Western blot. Data are expressed as mean ± SEM of five independent experiments (*n* = 5). ^#^ *p* < 0.01, as compared with the cells exposed to the indicated reagents.

Another member of MAPKs, JNK1/2 might also play an important role in the induction of HO-1 [28]. To evaluate the effect of JNK1/2 on HO-1 expression, we observed that pretreatment of HPAEpiCs with a JNK1/2 inhibitor (SP600125) concentration-dependently attenuated HO-1 protein and mRNA expression induced by MVS (Figure 5A,B). To ensure the role of JNK1/2 in HO-1 expression, as shown in Figure 5C, transfection with either JNK1 or JNK2 siRNA down-regulated the level of JNK1 or JNK2 protein and attenuated the MVS-induced HO-1 expression. Activation of JNK1/2 has been shown to upregulate Nrf2/HO-1 axis protecting against the oxidative impairment in RAW264.7 cells triggered by H_2_O_2_ [29]. Thus, we investigated whether phosphorylation of JNK1/2 was involved in the MVS-induced HO-1 expression. Our results showed that transfection with JNK1/2 siRNA knocked down the level of JNK1/2 protein and attenuated the MVS-stimulated phosphorylation of JNK1/2, but not PKCα and Pyk2 (Figure 5D). Further, MVS-stimulated phosphorylation of JNK1/2 was also attenuated by transfection with either PKCα or Pyk2 siRNA (Figure 5E). These results suggested that MVS-induced HO-1 expression is mediated through either PKCα/Pyk2/p38 MAPK or JNK1/2 in HPAEpiCs.

### 3.5. Upregulation of AP-1 Activity Is Mediated through PKCα/Pyk2/p38α MAPK or JNK1/2 Cascade

**Figure 6:**
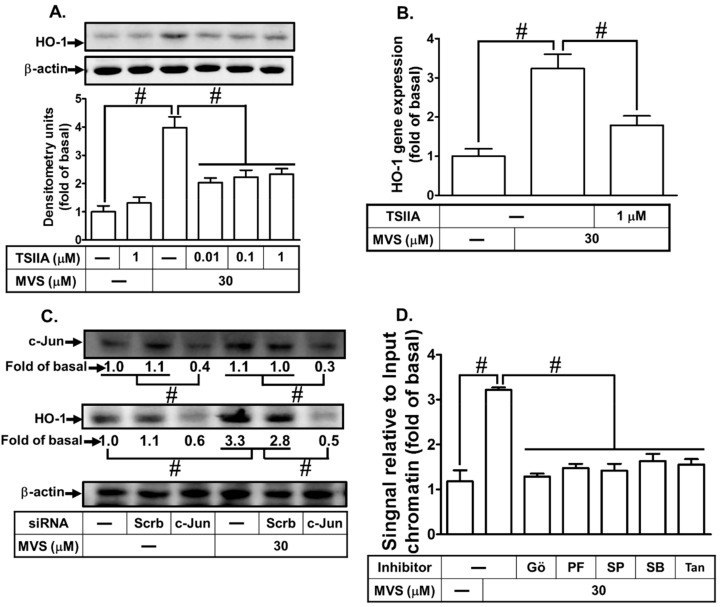

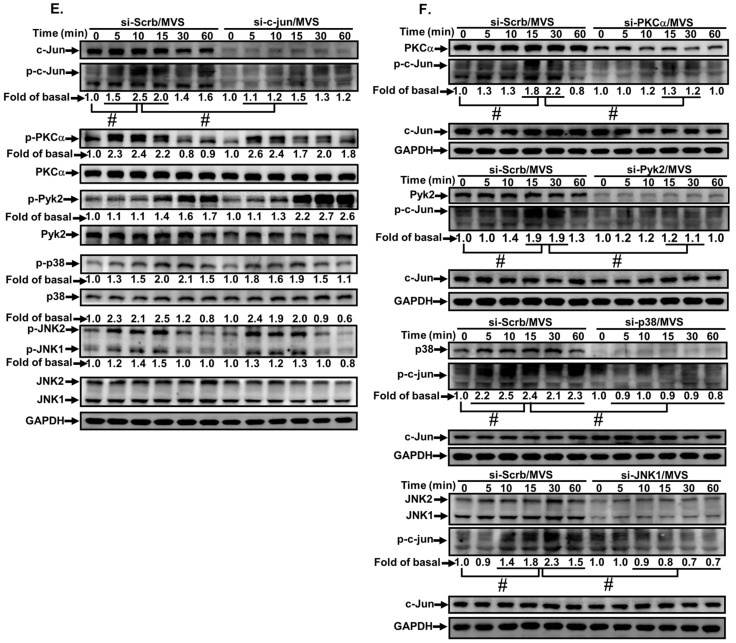
The involvement of PKCα/Pyk2/p38 MAPK or JNK1/2-dependent c-Jun/AP-1 activation in the MVS-induced HO-1 expression. (**A**) The cells were pretreated with various concentrations of Tanshinone IIA (0.01, 0.1, and 1 μM) for 1 h, and then incubated with vehicle or MVS for 24 h. The levels of HO-1 and β-actin protein expression were determined by Western blot. (**B**) The cells were pretreated with Tanshinone IIA (1 μM) for 1 h and then incubated with vehicle or MVS (30 μM) for 8 h. The levels of HO-1 mRNA were analyzed by real-time qPCR. (**C**) HPAEpiCs were transfected with scrambled (Scrb) or c-Jun siRNA, and then incubated with MVS (30 μM) for 24 h. The levels of c-Jun, HO-1, and β-actin protein expression were determined by Western blot. (**D**) Cells were pretreated without or with 10 μM Gö6983, 10 μM PF431396, 100 nM SB202190, 10 μM SP600125, or 1 μM Tanshinone IIA for 1 h and then stimulated by 30 μM MVS for 30 min. The levels of c-Jun binding to AP-1 region on the HO-1 promoter were detected by a SYBR green real-time qPCR. (**E**,**F**) HPAEpiCs were transfected with c-Jun, PKCα, Pyk2, p38α, or JNK1/2 siRNA, and then incubated with vehicle or MVS (30 μM) for the indicated time intervals. The levels of phospho-c-Jun, c-Jun, phospho-PKCα, PKCα, phospho-Pyk2, Pyk2, phospho-p38 MAPK, p38 MAPK, phospho-JNK1/2, JNK1/2, and GAPDH were determined by Western blot. Data are expressed as mean ± SEM of five independent experiments (*n* = 5). ^#^ *p* < 0.01, as compared with the cells exposed to the indicated reagents.

Activation of MAPKs could regulate phosphorylation of c-Jun which interacts with AP-1 binding sites on *hmox1* gene stimulated by various factors [17,19,20]. To clarify the roles of c-Jun/AP-1 in the MVS-induced HO-1 expression, an AP-1 inhibitor (Tanshinone IIA) was used for this purpose. As shown in Figure 6A,B, MVS-induced HO-1 protein and mRNA levels were significantly reduced by Tanshinone IIA in a concentration-dependent manner. Moreover, c-Jun has been recognized as a central subunit of AP-1 complexes [30]. To further investigate the role of c-Jun in MVS-mediated HO-1, as shown in Figure 6C, transfection with c-Jun siRNA knocked down the level of c-Jun protein and attenuated the MVS-induced HO-1 expression. Further, there are post-transcriptional modifications of c-Jun to regulate AP-1 activity involved in cellular functions [20,30]. To investigate whether activation of c-Jun/AP-1 binding site initiates HO-1 gene expression in HPAEpiCs, as shown in Figure 6D, MVS stimulated the interaction of c-Jun with AP-1 binding site on HO-1 promoter which was attenuated by pretreatment with Gö6983, PF431396, SB202190, SP600125, or Tanshinone IIA.

To determine whether MVS-stimulated c-Jun phosphorylation was mediated through PKCα/Pyk2/p38 MAPK- or JNK1/2-dependent cascade, we found that transfection with PKCα, Pyk2, p38 MAPK, or JNK1 siRNA attenuated phosphorylation of c-Jun stimulated by MVS (Figure 6E). Further, transfection with c-Jun siRNA only attenuated the phosphorylation of c-Jun and failed to inhibit the phosphorylation of PKCα, Pyk2, p38 MAPK, or JNK1/2 (Figure 6F). These results suggested that MVS-induced HO-1 expression is mediated through PKCα/Pyk2/p38 MAPK or JNK1/2-dependent AP-1 activation in HPAEpiCs.

## 4. Discussion

**Figure 7:**
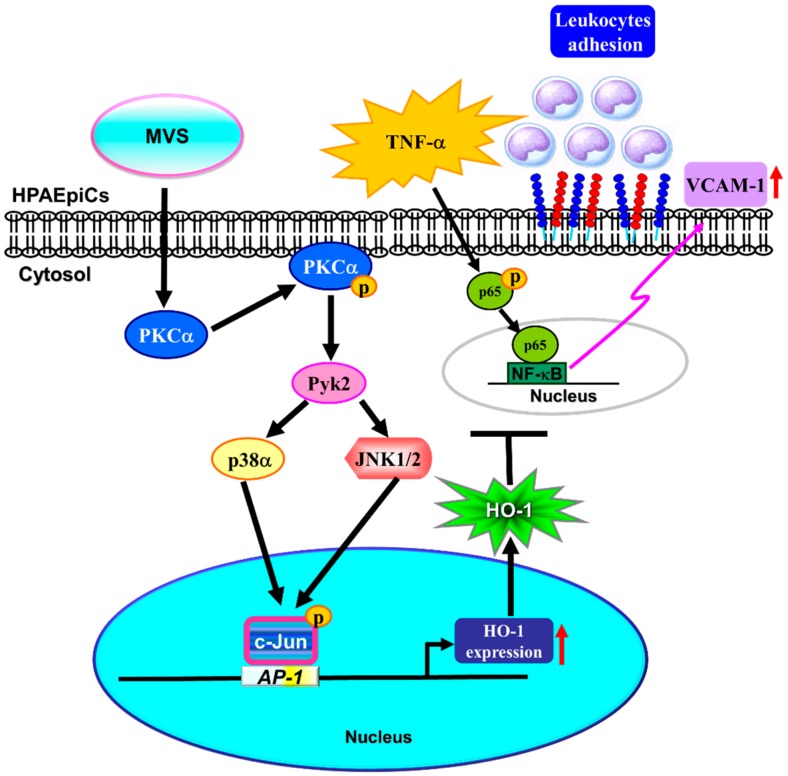
A schematic pathway for MVS-induced HO-1 expression protecting against the inflammatory responses in HPAEpiCs challenged with TNFα. MVS-induced HO-1 expression was mediated via PKCα/Pyk2/p38 MAPK or JNK1/2-dependent phosphorylation of c-Jun binding to AP-1 binding sites on HO-1 promoter that attenuated the TNFα-stimulated VCAM-1 expression and activation of NF-κB p65.

Administration of MVS has been shown to markedly reduce S1P-induced the cell migration of tracheal smooth muscle cells in the airway inflammatory model [11]. MVS could also induce the expression of HO-1 in murine RAW264.7 macrophages [10]. Further, up-regulation of HO-1 might exert as a potential therapeutic strategy for the treatment of airway inflammatory diseases [21,31]. However, the detail molecular mechanisms and anti-inflammatory function of MVS-induced HO-1 expression are not fully defined in HPAEpiCs. In this study, we observed that pretreatment with MVS reduced the TNFα-induced VCAM-1 expression through up-regulation of HO-1. MVS-mediated responses were attenuated by pretreatment with pharmacological inhibitors (GF109203X, Gö6983, PF431396, SB202190, SP600125, or Tanshinone IIA) and transfection with siRNA (PKCα, Pyk2, p38α, JNK1, or c-Jun). Here, our study demonstrated that MVS-induced HO-1 is mediated via activation of PKCα/Pyk2/MAPK (p38 MAPK or JNK1/2)/c-Jun/AP-1 pathway and protects against the inflammatory responses in HPAEpiCs challenged with TNFα (Figure 7).

TNFα is involved in the process of infection and immune responses [32]. TNFα stimulates NF-κB p65-dependent pathway mediated pathogenesis of different diseases [33,34]. NF-κB p65 represents one of the inducible transcription factors that play an important role in the expression of multiple genes involved in the processes of inflammatory responses [24]. Moreover, NF-κB activated by various signaling mechanisms could be responsible for the production of pro-inflammatory mediators, contributing to the development of inflammatory diseases [35]. Therefore, targeting the NF-κB p65 represents an attractive approach for anti-inflammatory therapies [3]. Overexpression of HO-1 can down-regulate the TNFα-mediated undergoing programmed apoptosis through the phosphorylation of NF-κB p65/RelA [1,36]. HO-1 plays an important role in the treatment of various diseases due to its pleiotropic effect on organs protection [8]. Moreover, several pharmacological compounds have been proved to induce HO-1 expression [37]. This induction of HO-1 is, at least in part, responsible for the perceived therapeutic efficacy of these pharmacological compounds [38]. Statins, HMG-CoA reductase inhibitors, have not only cholesterol-lower function but also pleiotropic anti-inflammatory, anti-oxidant, and anti-cancer effects [39]. Statins have a potential benefit which could arise from their effects attenuating the expression of inflammatory cytokines, as well as modulation of both innate and adaptive immune systems [40]. These findings are in line with our results indicating that MVS-induced HO-1 expression protects against the NF-κB p65-dependent VCAM-1 expression induced by TNFα. This inhibitory effect was revered by SnPPIX (an HO-1 enzyme inhibitor) in HPAEpiCs. Therefore, our results suggested that MVS-mediated phase II antioxidant enzyme HO-1 expression could be a potential therapeutic strategy for the pulmonary inflammatory diseases.

PKC is a family of Ser/Thr kinases that play an essential role in the regulation of multi-signal transductions for cellular functions [41]. Multiple isozymes of PKC can be categorized into three groups, including conventional (α, βI, βII, and γ), novel (β, ε, η, and θ), and atypical (ζ and ι) [42]. Among these isoforms, PKCα is one of the most prominent modulators of HO-1 expression, which improve mitochondrial dynamics via increasing the expression of fusion proteins [13]. The effect of PKCα was confirmed by our results indicating that MVS-induced HO-1 expression was mediated through PKCα membrane translocation and phosphorylation, which were inhibited by Gö6983 or transfection with its siRNA. Furthermore, Pyk2 has been shown to be a downstream component activated by PKC [14]. Our previous studies demonstrated that activation of Pyk2 is involved in HO-1 expression and protecting against lung inflammatory responses [15,21]. The results in current study confirmed that Pyk2 played a key role in the MVS-induced HO-1 expression, which was reduced by PF431396 or transfection with Pyk2 siRNA. Phosphorylation of Pyk2-mediated HO-1 was also attenuated by transfection with either PKCα or Pyk2 siRNA. In contrast, transfection with Pyk2 siRNA had no effect on activation of PKCα, suggesting that Pyk2 is a downstream component of PKCα. These results verified that the mechanisms underlying MVS-induced HO-1 expression is mediated through PKCα-dependent Pyk2 phosphorylation in HPAEpiCs.

MAPKs are involved in the HO-1 expression induced by diverse stimuli [43,44]. Activation p38 MAPK upregulates Nrf2/Egr1 translocating to nucleus and HO-1/GCLc expression [45]. Our previous study also showed that activation of p38 MAPK could protect against inflammatory responses of lung challenged by LPS through upregulation of antioxidant enzyme HO-1 [6]. In addition, statins-induced HO-1 gene transcription is mediated through p38 MAPK activation in macrophages [10]. Expression of HO-1 mediated through JNK1/2 signaling could attenuate cell death in chronic cholestatic liver disease and sepsis [26]. Activation of ERK1/2-dependent Nrf2 signaling pathway might defend against oxidative stress [46]. These studies have indicated that HO-1 expression is mediated through the MAPKs signaling pathway. In the present study, we found that p38 MAPK and JNK1/2 were involved in the MVS-induced HO-1 expression in HPAEpiCs. The induction of HO-1 was attenuated by their pharmacological inhibitors or transfection with respective siRNAs. In particular, phosphorylation of p38 MAPK and JNK1/2 was attenuated by transfection with either PKCα or Pyk2 siRNA. Therefore, our results demonstrated that p38 MAPK and JNK1/2 were downstream components of PKCα/Pyk2, leading to HO-1 expression in HPAEpiCs treated with MVS.

AP-1 is a heterodimer protein families composed of Jun (c-Jun, Jun B, and Jun D), Fos (c-Fos, FosB, Fra1, and Fra2), and ATF (activating transcription factor) [47]. AP-1 families are one of the most important and the best-studied regulators of the cellular stresses [48]. The transcription factors and the regulatory regions that are responsible for the induction of HO-1 gene by MVS have not been elucidated. Therefore, AP-1 modulates cellular functions through the expression of various target proteins, such as HO-1, that displays anti-inflammatory and anti-oxidant effects [19]. In particular, serval studies have identified various AP-1 binding sites on a *hmox1* gene and verified their functions in response to HO-1 inducers [19,20]. These studies have established the roles of tyrosine kinase and JNK1/2 in the phosphorylation of c-Jun leading to activation of AP-1 [17]. Our results indicated that MVS-induced HO-1 expression was, at least in part, mediated through c-Jun/AP-1 activation which was attenuated by pretreatment with Tanshinone IIA or transfection with c-Jun siRNA. Indeed, ChIP assay results suggested that MVS stimulates the interaction of c-Jun and AP-1 binding site on HO-1 promoters, which is attenuated by the pharmacological inhibitors including Gö6983, PF431396, SB202190, SP600125, and Tanshinone IIA. These results suggested that AP-1 is required for the MVS-induced HO-1 expression mediated through activation of PKCα/Pyk2/p38 MAPK or JNK1/2-dependent cascade.

In the respiratory system, the pulmonary alveolar epithelium is composed of Type I and Type II alveolar epithelial cells. Type I cells comprise approximately 40% of the alveolar epithelium and form the epithelial component of the thin air–blood barrier and are responsible for the gas exchange of CO_2_/O_2_ that takes place. Type II cells comprise 60% of the alveolar epithelium and 15% of the peripheral lung cells. [49]. The biological activity of the primary human pulmonary Type II cells producing SP-C, cytokines, and intercellular adhesion molecule-1 is vigorous in response to stimulation with TNF-α [50]. In our study, HPAEpiCs contained Type I and II, which are not recommended for expanding or long-term cultures since the cells differentiate to become Type I cells immediately after plating. Type I alveolar epithelial cells do not proliferate in culture. Thus, only the passages of HPAEpiCs from 4 to 6 were used throughout this study.

## 5. Conclusions

In summary, based on the above results and literatures, our results demonstrated that MVS-induced HO-1 expression might play a protective role in pulmonary inflammatory responses, which is mediated through phosphorylation of PKCα/Pyk2-dependent p38 MAPK or JNK1/2 cascade to enhance c-Jun binding with AP-1 region, and finally leading to HO-1 expression in HPAEpiCs. Although several studies have supported the potentially anti-inflammatory properties of statins, the detailed mechanisms should be elucidated in the future. These findings could expand the application of HMG-CoA inhibitors as a potential intervention for the prevention or treatment of lung inflammatory diseases.

## References

[1] Gozzelino R., Jeney V., Soares M.P. (2010). Mechanisms of Cell Protection by Heme Oxygenase-1. Annu. Rev. Pharmacol. Toxicol..

[2] Chen L., Deng H., Cui H., Fang J., Zuo Z., Deng J., Li Y., Wang X., Zhao L. (2018). Inflammatory responses and inflammation-associated diseases in organs. Oncotarget.

[3] Giridharan S., Srinivasan M. (2018). Mechanisms of NF-kappaB p65 and strategies for therapeutic manipulation. J. Inflamm. Res..

[4] Yang H., Chen B., Zhao Z., Zhang L., Zhang Y., Chen J., Zhang X., Zhang X., Zhao L. (2018). Heme oxygenase-1 exerts pro-apoptotic effects on hepatic stellate cells in vitro through regulation of nuclear factor-kappaB. Exp. Ther. Med..

[5] Zhang L.J., Zhang Z.L., Liu B.J., Jin Y.L., Tian Y., Xin Y., Duan Z.J. (2017). The Protective Effect of Heme Oxygenase-1 against Intestinal Barrier Dysfunction in Cholestatic Liver Injury Is Associated with NF-jB Inhibition. Mol. Med..

[6] Cho R.L., Lin W.N., Wang C.Y., Yang C.C., Hsiao L.D., Lin C.C., Yang C.M. (2018). Heme oxygenase-1 induction by rosiglitazone via PKCalpha/AMPKalpha/p38 MAPKalpha/SIRT1/PPARgamma pathway suppresses lipopolysaccharide-mediated pulmonary inflammation. Biochem. Pharmacol..

[7] Chang M., Xue J., Sharma V., Habtezion A. (2015). Protective role of hemeoxygenase-1 in gastrointestinal diseases. Cell. Mol. Life Sci..

[8] Mehta J., Rayalam S., Wang X. (2018). Cytoprotective Effects of Natural Compounds against Oxidative Stress. Antioxidants.

[9] Waza A.A., Hamid Z., Ali S., Bhat S.A., Bhat M.A. (2018). A review on heme oxygenase-1 induction: Is it a necessary evil. Inflamm. Res..

[10] Chen J.C., Huang K.C., Lin W.W. (2006). HMG-CoA reductase inhibitors upregulate heme oxygenase-1 expression in murine RAW264.7 macrophages via ERK, p38 MAPK and protein kinase G pathways. Cell. Signal..

[11] Hsu C.K., Lin C.C., Hsiao L.D., Yang C.M. (2015). Mevastatin ameliorates sphingosine 1-phosphate-induced COX-2/PGE2-dependent cell migration via FoxO1 and CREB phosphorylation and translocation. Br. J. Pharmacol..

[12] Gould C.M., Newton A.C. (2008). The life and death of protein kinase C. Curr. Drug Targets.

[13] Li X., Zhang Y., Yu J., Mu R., Wu L., Shi J., Gong L., Liu D. (2018). Activation of protein kinase C-alpha/heme oxygenase-1 signaling pathway improves mitochondrial dynamics in lipopolysaccharide-activated NR8383 cells. Exp. Ther. Med..

[14] Bartos J.A., Ulrich J.D., Li H., Beazely M.A., Chen Y., Macdonald J.F., Hell J.W. (2010). Postsynaptic clustering and activation of Pyk2 by PSD-95. J. Neurosci..

[15] Lin C.C., Chiang Y.C., Cho R.L., Lin W.N., Yang C.C., Hsiao L.D., Yang C.M. (2018). Up-regulation of PYK2/PKCalpha-dependent haem oxygenase-1 by CO-releasing molecule-2 attenuates TNF-alpha-induced lung inflammation. Br. J. Pharmacol..

[16] Lin C.C., Yang C.C., Hsiao L.D., Chen S.Y., Yang C.M. (2017). Heme Oxygenase-1 Induction by Carbon Monoxide Releasing Molecule-3 Suppresses Interleukin-1beta-Mediated Neuroinflammation. Front. Mol. Neurosci..

[17] Harada H., Sugimoto R., Watanabe A., Taketani S., Okada K., Warabi E., Siow R., Itoh K., Yamamoto M., Ishii T. (2008). Differential roles for Nrf2 and AP-1 in upregulation of HO-1 expression by arsenite in murine embryonic fibroblasts. Free Radic. Res..

[18] Park J.S., Kim H.S. (2014). Regulation of hemeoxygenase-1 gene expression by Nrf2 and c-Jun in tertiary butylhydroquinone-stimulated rat primary astrocytes. Biochem. Biophys. Res. Commun..

[19] Lee P.J., Camhi S.L., Chin B.Y., Alam J., Choi A.M. (2000). AP-1 and STAT mediate hyperoxia-induced gene transcription of heme oxygenase-1. Am. J. Physiol. Lung Cell. Mol. Physiol..

[20] Alam J., Cook J.L. (2007). How many transcription factors does it take to turn on the heme oxygenase-1 gene?. Am. J. Respir. Cell Mol. Biol..

[21] Cho R.L., Yang C.C., Tseng H.C., Hsiao L.D., Lin C.C., Yang C.M. (2018). Haem oxygenase-1 up-regulation by rosiglitazone via ROS-dependent Nrf2-antioxidant response elements axis or PPARgamma attenuates LPS-mediated lung inflammation. Br. J. Pharmacol..

[22] Cho R.L., Yang C.C., Lee I.T., Lin C.C., Chi P.L., Hsiao L.D., Yang C.M. (2016). Lipopolysaccharide induces ICAM-1 expression via a c-Src/NADPH oxidase/ROS-dependent NF-kappaB pathway in human pulmonary alveolar epithelial cells. Am. J. Physiol. Lung Cell. Mol. Physiol..

[23] Lin C.C., Lin W.N., Cho R.L., Yang C.C., Yeh Y.C., Hsiao L.D., Tseng H.C., Yang C.M. (2020). Induction of HO-1 by mevastatin mediated via a Nox/ROS-dependent c-Src/PDGFRα/PI3K/Akt/Nrf2/ARE cascade suppresses TNF-α-induced lung inflammation. J. Clin. Med..

[24] Liu T., Zhang L., Joo D., Sun S.C. (2017). NF-kappaB signaling in inflammation. Signal. Transduct. Target. Ther..

[25] Fang Z., Xu A., Wu L., Hei T.K., Hong M. (2016). The role of protein kinase C alpha translocation in radiation-induced bystander effect. Sci. Rep..

[26] Song Y.J., Zong Z.M., Liu H.Z., Mukasa R., Pei D.S., Mou J., Wen X.R., Liu Z.A., Wei X.Y. (2012). Heme oxygenase-1 regulates the JNK signaling pathway through the MLK3-MKK7-JNK3 signaling module in brain ischemia injury. Brain Res..

[27] Yao X., Lu B., Lu C., Bai Q., Yan D., Wu Y., Hong Z., Xu H. (2017). Solanesol induces the expression of heme oxygenase-1 via p38 and Akt and suppresses the production of proinflammatory cytokines in RAW264.7 cells. Food Funct..

[28] Xu J., Li J.S., Wang J.H., Chi Y.C., Zhang K., Cui R. (2016). Heme oxygenase-1 protects H2O2-insulted glomerular mesangial cells from excessive autophagy. Mol. Med. Rep..

[29] Sebastian V.P., Salazar G.A., Coronado-Arrazola I., Schultz B.M., Vallejos O.P., Berkowitz L., Alvarez-Lobos M.M., Riedel C.A., Kalergis A.M., Bueno S.M. (2018). Heme Oxygenase-1 as a Modulator of Intestinal Inflammation Development and Progression. Front. Immunol..

[30] Haodang L., Lianmei Q., Ranhui L., Liesong C., Jun H., Yihua Z., Cuiming Z., Yimou W., Xiaoxing Y. (2019). HO-1 mediates the anti-inflammatory actions of Sulforaphane in monocytes stimulated with a mycoplasmal lipopeptide. Chem. Biol. Interact..

[31] Lin C.C., Hsiao L.D., Cho R.L., Yang C.M. (2019). CO-Releasing Molecule-2 Induces Nrf2/ARE-Dependent Heme Oxygenase-1 Expression Suppressing TNF-alpha-Induced Pulmonary Inflammation. J. Clin. Med..

[32] Duque G.A., Descoteaux A. (2014). Macrophage cytokines: Involvement in immunity and infectious diseases. Front. Immunol..

[33] Hayden M.S., Ghosh S. (2014). Regulation of NF-kappaB by TNF family cytokines. Semin. Immunol..

[34] Hoesel B., Schmid J.A. (2013). The complexity of NF-kappaB signaling in inflammation and cancer. Mol. Cancer.

[35] Hou J., Ma T., Cao H., Chen Y., Wang C., Chen X., Xiang Z., Han X. (2018). TNF-alpha-induced NF-kappaB activation promotes myofibroblast differentiation of LR-MSCs and exacerbates bleomycin-induced pulmonary fibrosis. J. Cell. Physiol..

[36] Huang J., Guo P.X., Ma D., Lin X.J., Fang Q., Wang J. (2016). Overexpression of heme oxygenase-1 induced by constitutively activated NF-kappa B as a potential therapeutic target for activated B-cell-like diffuse large B-cell lymphoma. Int. J. Oncol..

[37] Li C., Hossieny P., Wu B.J., Qawasmeh A., Beck K., Stocker R. (2007). Pharmacologic induction of heme oxygenase-1. Antioxid. Redox Signal..

[38] Durante W. (2010). Targeting heme oxygenase-1 in vascular disease. Curr. Drug Targets.

[39] Jang H.J., Hong E.M., Kim M., Kim J.H., Jang J., Park S.W., Byun H.W., Koh D.H., Choi M.H., Kae S.H. (2016). Simvastatin induces heme oxygenase-1 via NF-E2-related factor 2 (Nrf2) activation through ERK and PI3K/Akt pathway in colon cancer. Oncotarget.

[40] Quist-Paulsen P. (2010). Statins and inflammation: An update. Curr. Opin. Cardiol..

[41] Jiang L.J., Zhang S.M., Li C.W., Tang J.Y., Che F.Y., Lu Y.C. (2017). Roles of the Nrf2/HO-1 pathway in the anti-oxidative stress response to ischemia-reperfusion brain injury in rats. Eur. Rev. Med. Pharmacol. Sci..

[42] Newton A.C. (2018). Protein kinase C: Perfectly balanced. Crit. Rev. Biochem. Mol. Biol..

[43] Osaki L.H., Gama P. (2013). MAPKs and signal transduction in the control of gastrointestinal epithelial cell proliferation and differentiation. Int. J. Mol. Sci..

[44] Chi P.L., Lin C.C., Chen Y.W., Hsiao L.D., Yang C.M. (2015). CO Induces Nrf2-Dependent Heme Oxygenase-1 Transcription by Cooperating with Sp1 and c-Jun in Rat Brain Astrocytes. Mol. Neurobiol..

[45] Koyani C.N., Kitz K., Rossmann C., Bernhart E., Huber E., Trummer C., Windischhofer W., Sattler W., Malle E. (2016). Activation of the MAPK/Akt/Nrf2-Egr1/HO-1-GCLc axis protects MG-63 osteosarcoma cells against 15d-PGJ2-mediated cell death. Biochem. Pharmacol..

[46] Park J.Y., Kang K.A., Kim K.C., Cha J.W., Kim E.H., Hyun J.W. (2013). Morin Induces Heme Oxygenase-1 via ERK-Nrf2 Signaling Pathway. J. Cancer Prev..

[47] Gazon H., Barbeau B., Mesnard J.M., Peloponese J.M. (2017). Hijacking of the AP-1 Signaling Pathway during Development of ATL. Front. Microbiol..

[48] Dennery P.A. (2014). Signaling function of heme oxygenase proteins. Antioxid. Redox Signal..

[49] Fehrenbach H. (2001). Alveolar epithelial type II cell: Defender of the alveolus revisited. Respir. Res..

[50] Mao P., Wu S., Li J., Fu W., He W., Liu X., Slutsky A.S., Zhang H., Li Y. (2015). Human alveolar epithelial type II cells in primary culture. Physiol. Rep..

